# Active Ageing in CIS Countries: Semantics, Challenges, and Responses

**DOI:** 10.1155/2013/261819

**Published:** 2013-01-03

**Authors:** Alexandre Sidorenko, Asghar Zaidi

**Affiliations:** ^1^European Centre for Social Welfare Policy and Research, Berggasse 17, 1090 Vienna, Austria; ^2^Centre for Research on Ageing, Social Sciences, Southampton University, Murray Building, Southampton SO17 1BJ, UK

## Abstract

Although the CIS countries are connected together by the legacy of breaking away from the Soviet Union, they have had a distinctive transition course and are rather diverse in terms of the population ageing challenges and policy responses in place. The commonality is that a comprehensive national strategy on ageing is lacking, and many of necessary reforms were put aside owing to political uncertainties, lack of societal consensus, and financial instability. The notion of active ageing is associated with the term “accelerated ageing,” which is understood to be an individual living a life under harsh living conditions or a society experiencing rapid increases in the relative number of older persons, and therefore it carries a negative connotation. Yet, in the same spirit as the European Year for Active Ageing and Solidarity between Generations 2012, the CIS countries have initiated sectoral programmes towards enhancing employment of older workers, social participation of older people in the society in a wider sense and also measures promoting health and independent living of older persons.

## 1. Introduction

The term active ageing has now occupied a central place in the international discourse on policy on ageing, particularly in the EU-27 countries which have been observing the European Year for Active Ageing and Solidarity between Generations 2012 (EY2012). It is reasonable to expect that the EY2012 would contribute to raising awareness on the European as well as international discourse on active ageing and also in informing the policy interventions required. Meanwhile, the impact of the EY2012 can be extended beyond the twenty-seven EU countries and some essential lessons can also be shared internationally, including in the immediate EU neighbourhood, the Commonwealth of Independent States (CIS) countries. 

This paper is about active ageing policy discourse in the CIS countries, and is organised in six sections. After this introduction, Section 2 outlines the specific patterns of the active ageing concept used in the international policy frameworks, in the CIS countries as well as in other parts of the world.  Section 3 presents the demographic outlook of these countries and highlights the diversity across CIS countries with respect to the phenomenon of population ageing.  Section 4 reports on the differential extent of challenges faced by these countries, in terms of employment, social participation and capacity to live a healthy and independent life, in line with the three dimensions of the EY2012.  Section 5 describes the policy approaches adopted across these countries.  Section 6 provides a concluding summary. 

## 2. Definitions and Regional Semantics

### 2.1. Definitions and Policy Frameworks

The various definitions of active ageing as a policy concept and a policy framework are of multidimensional (multifaceted) nature. In its 2002 milestone publication, World Health Organization (WHO) defines active ageing as “the process of optimizing opportunities for health, participation and security in order to enhance quality of life as people age” [1]. This 2002 WHO policy framework underlines that active ageing aims to extend healthy life expectancy and have the overall objective of improving the quality of life for all people as they age (especially those who are frail, disabled, and in need of care). It would therefore be pertinent to broaden the scope of active ageing dimensions by including, along with the productive (both remunerated and nonremunerated) work, the following activities and leisure: housework; active leisure (hobbies, sports, travel, creative activities, education, and social contacts); home-based and family-related leisure; and everyday physical and cognitive activities (such as solving crosswords and reading). The two latter areas are of particular relevance to frail older persons and older persons with disabilities [2].

The WHO Active Ageing publication has been a contribution to the Second World Assembly on Ageing (WAA), 2002. The major outcome of this WAA has been the Madrid International Plan of Action on Ageing (MIPAA) [3]. Although MIPAA does not contain an elaborated definition of active ageing, the Political Declaration signed at the end of the 2nd WAA emphasizes the two essential elements directly relevant to active ageing policy discourse: the empowerment of older persons and the promotion of their full participation. Moreover, MIPAA contains several policy recommendations pertinent to active ageing. These recommendations are concerned with the active participation in society and development (priority issue 1 of the first priority direction of MIPAA), access to knowledge, education and training (priority issue 4 of the first priority direction), and health promotion and well-being throughout life (priority issue 1 of the second priority direction). 

OECD defines active ageing somewhat narrowly as “the capacity of people, as they grow older, to lead productive lives in the society and the economy.” Thus the main focus of the OECD policy concept related to active ageing is to promote productive activities of older people [4]. It misses out on putting emphasis on health enhancing activities and capacity for autonomous living. 

One of the most recent definitions of active ageing has been offered for the EU Year for Active Ageing and Solidarity between Generations 2012: “active ageing means growing old in good health and as a full member of society, feeling more fulfilled in our jobs, more independent in our daily lives and more involved as citizens” [5]. A more comprehensive definition comes from the background research work undertaken for the active ageing index, constructed for the European Commission and the UN Economic Commission for Europe (UNECE), in the framework of the 10th anniversary of the 2nd WAA, the 2nd cycle of review and appraisal of the implementation of MIPAA and its European Regional Implementation Strategy (RIS), and the EY2012: “Active ageing refers to the social ageing phenomenon in which, with rising life expectancy on average, people are expected and allowed to continue to participate longer in the formal labour market as well as in other unpaid productive activities (such as care provision to family members and volunteering) and live healthy, independent and autonomous lives in their older ages.” The same report highlights that active ageing is also important as a determining factor of the quality of life of older people and the sustainability of public welfare systems: “in view of diversities across European countries and across subgroups, it is vital to assess not just how countries and subgroups fare in terms of actual experiences of active ageing but also measure the unrealised potential of older people that can still be tapped to improve their quality of life and to make public welfare systems more sustainable” [6]. 

The cumulative nature of definitions of “active ageing” allows for a broad interpretation of the corresponding policy frameworks. The 2002 WHO policy framework implies policy action in three areas: health, participation, and security.

- “Health” is referred to as physical, mental, and social well-being, following the WHO definition of health.
- “Participation” is in turn understood as a multi-faceted array of activities by older persons in social, economic, cultural, spiritual, and civic affairs, in addition to their participation in the labour force.
- “Security” is concerned with the access of older persons to physical and social environment; income security; and (when applicable) the securing of dignified work.

The UNECE identifies three areas of policy actions on active ageing, though put somewhat differently from the three areas defined by WHO but essentially capturing the three important domains: labour market participation, social integration and health. 

EY2012 also seeks to promote active ageing in three broadly defined areas: employment; participation in society; independent living. Measures in the area of employment aim at creating better opportunities and employability for older workers; measures in the area of participation are to be focused on combating the social exclusion of older people by fostering their active participation in the society (by encouraging voluntary activities and support for informal carers); measures in the area of independent living should encourage healthy ageing and independent self-reliant living by emphasizing a preventive approach in health and social care, making transport more accessible, and making the environment more age friendly [7].

The differences in interpreting the meaning of “active ageing” are noticeable also in the definitions adopted at the national level. For example, in the Australian context the proposed five elements of active ageing also include financial security, in addition to being active socially, mentally, and physically, and workforce participation [8]. The Public Health Agency of Canada, on the other hand, stipulates that active ageing can be enabled by measures that support living in a safe home with adequate nutrition, having appropriate transportation and a social network, and having access to information, health, and social services [9].

Thus, the diversity of the meaning of the term “active ageing” makes the goal of identifying common grounds in discussions and in comparing policy implementation and research more complex, both internationally and nationally [8]. 

The definition used in the CIS countries is not devoid of similar diversities and complexities. For the analysis of the active ageing approaches in policy actions on ageing in CIS countries, a three-dimensional framework is considered most insightful (also to be used in this paper). This framework is based on the three dimensions of promoting active ageing in the framework of the EY2012, slightly modified [7, 10]. The three policy dimensions include *employment*, *social participation in society*, and *independent* and *autonomous living* (covering preventive health care, accessible transport, and age-friendly environment). Such a framework would allow us to better capture information available on policy measures aimed towards promoting active ageing, as well as identify the most significant gaps in national policies on ageing in the CIS countries. 

### 2.2. Semantics in CIS Countries

At the outset, it would be worthwhile to note that the term “active ageing” is practically of little use in the CIS countries, mainly for the fact that it can have a negative connotation in many (Slavic) languages of the CIS countries. The notion “active ageing” might almost unconsciously turn on an image of someone who has become old too fast by accelerating through his/her life course. Such an image originates from the recognition that citizens of many CIS countries belong to a society where *accelerated ageing* prevails at both individual and societal level.

- At the individual level, the accelerated (i.e., “active”) ageing can be attributed to living one's life under harsh living and working conditions, environmental threats (e.g., Chernobyl disaster), and poor provision of health and social services, as was the experience of many individuals during the transition period.
- At the societal level, the accelerated ageing in the CIS countries implies rapid increase in the relative number of older persons owing to low fertility (major cause of population ageing everywhere in the world), high mortality of younger people (a disastrous feature of many CIS countries), and, in some countries, such as the Republic of Moldova, mass emigration of young labourers.

A negative view of ageing in general, and the active ageing in particular, can be attributed to the legacy of the recent past and the hardships of the continuing and still incomplete transition from the communist past in many countries. Such a view might have been echoed in the recent survey of the Eurobarometer [7], which reflected more negative views of ageing (such as older persons being a burden on the society) by the citizens in the “new” Central and Eastern European member countries of the European Union as compared to the citizens of its first fifteen Western and Northern European member countries. These new member states used to belong until 1989 to the group of “socialist” countries and thus they share a legacy of the recent past with the people of the CIS countries. 

 The distinctive feature of activity by constraint and not by choice should also be kept in mind: low pensions for many pensioners force them to look for employment and thus additional income, and they often rely on activities such as small trade, and cottage agriculture [11]. As another example of regional semantics in CIS countries, the age beyond the retirement is often called the “age of working incapacity,” and people over the age of retirement are referred to as people “over the working age” [12]. 

The above considerations do not of course imply that the significance of active life style in older ages and throughout the life course is unknown or undesirable in the CIS countries. In terms of semantics, more acceptable term has been *active longevity* [13, 14] instead of active ageing. That said, we consider the notion of “active longevity” as a synonym of active ageing; therefore, in this paper we continue to use the term active ageing as prevailing in the western scientific and popular literature. 

## 3. Demographic Outlook 

**Figure 1:**
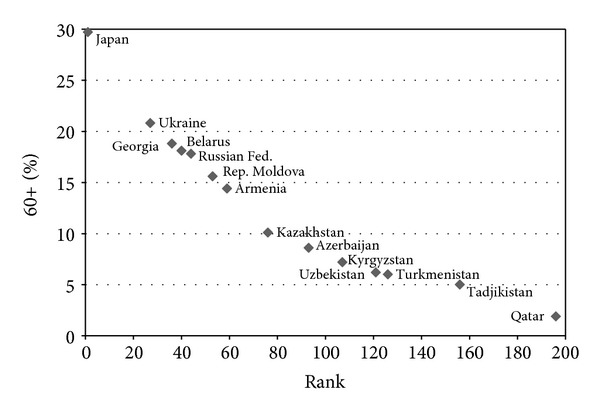
Ranking CIS countries by percentage of population aged 60+, 2009 (source: [15]).

While linked geopolitically, the CIS is a loose association of countries and they are undoubtedly very heterogeneous. This heterogeneity is particularly seen in terms of ageing of populations of these countries. Ukraine, for instance, is already among the fastest ageing countries of the world, along with the majority of other European countries. By using the indicator, the percentage of population aged 60 years or over, Ukraine in 2009 was ranked twenty-seventh, Georgia thirty-sixth, Belarus fortieth, among the 196 countries of the world (see Figure 1). In the countries of the European Union (EU), the percentage of population aged 60 years or over was in 2009 significantly higher. Among the ten most rapidly ageing countries of the world, eight were members of the EU; seventeen EU member states belonged to the group of the twenty oldest countries of the world; twenty-two EU member States were among the thirty world countries with the fastest ageing of populations. Five EU countries occupied ranks from thirty-fifth (Luxemburg) to fifty-second (Ireland). 

In the same year by this indicator, Kyrgyzstan was on the one hundred-seventh position, Uzbekistan on one hundred twenty-first, Turkmenistan on one hundred twenty-sixth, and Tajikistan was one hundred fifty-sixth [15].

**Figure 2:**
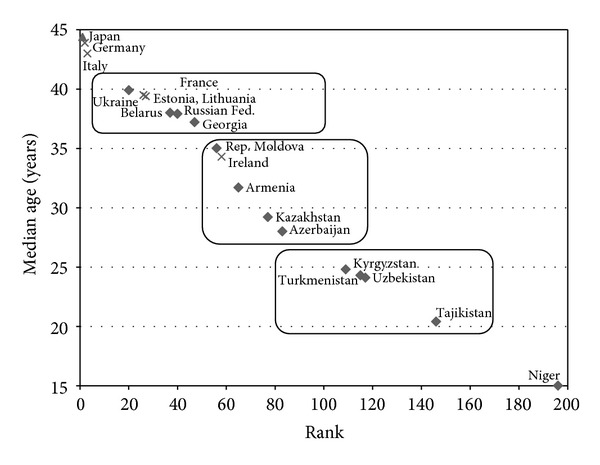
Ranking CIS countries by median age of population, 2009 (source: [15]).

By the median age indicator, Ukraine in 2009 was among the top thirty countries, and Belarus and Russian Federation among the top forty, while Kyrgyzstan, Tajikistan, Turkmenistan, and Uzbekistan were among the countries of the world on the other end of the spectrum of population ageing phenomenon (see Figure 2). 

On the basis of the above two indicators, the CIS countries occupy a wide space in a continuum of the demographic transition between Japan and Qatar, in case of the proportion of 60+ population, or Niger in case of the median age. At the same time, by the indicator of median age, one can group the CIS countries in three clusters: the first cluster of countries with the median age above 35 years (Belarus, Georgia, Russian Federation, and Ukraine); the second cluster with the median age between 25 and 35 years (Armenia, Azerbaijan, Kazakhstan, and Republic of Moldova); and the third cluster - with the median age below 25 years (Kyrgyzstan, Tajikistan, Turkmenistan, and Uzbekistan).

All EU countries in 2009 had their median age above 35 years thus belonging to the first cluster together with the four CIS countries. Moreover, six of them, Germany, Italy, Finland, Bulgaria, Austria, and Slovenia, were in the group of the ten countries with the highest median age between 44.4 and 41.4 years. Ireland with the median age of 34 was ranked 58th (Table 1). 

**Figure 3:**
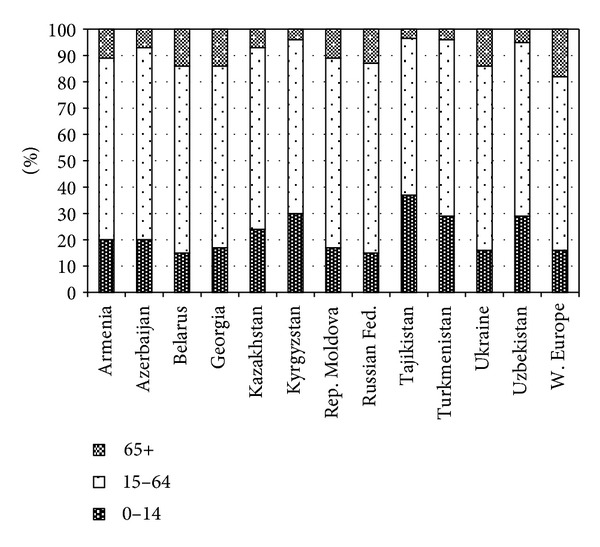
Age structure of population in CIS and Western European Countries, 2010. Note: W. (Western) Europe: Austria, Belgium, France, Germany, Liechtenstein*, Luxemburg, Monaco*, Netherlands, Switzerland* (*non-EU member) (source: [16]).

Significant heterogeneity of the CIS countries can also be noted in the age structure of their populations (Figure 3). The highest proportion of children (0–14 years old) in 2010 was in Tajikistan (37%) followed by Kyrgyzstan (30%) and Turkmenistan and Uzbekistan (29% each), and the lowest values of this indicator were in Belarus and the Russian Federation (15% in each country) followed by Ukraine (16%). In the group of the Western European countries [16], which includes 6 EU member states (Austria, Belgium, France, Germany, Luxemburg, and the Netherlands), the average proportion of children was 16%, the same value as in Ukraine, and slightly higher than in Belarus and the Russian Federation. 

The highest proportion of persons at the age of 65 years and over was in Belarus, Georgia, and Ukraine (14% in each country) followed by the Russian Federation (13%). The lowest is observed in Tajikistan (3.5%), followed by Kyrgyzstan and Turkmenistan (4% in each country) and Uzbekistan (5%). In the Western European countries the corresponding figure was higher −18%; with the highest value in Germany −20.4% [16]. 

The large and highly heterogeneous population group of persons at the age between 15 and 64 is often referred as the “working-age population” [17]. Among the CIS countries, the largest proportion of persons at this age is in Azerbaijan (73%) followed by the Republic of Moldova and the Russian Federation (both are at 72%), and Belarus (71%). The lowest proportion of persons of this age is in Tajikistan (59.5%) owing to a relatively higher proportion of children in the population of this country. Close figures are in the three other Central Asian countries: Kyrgyzstan and Uzbekistan (66% in each) and Turkmenistan (67%). The Western European countries have the same proportion of the working age population as Kyrgyzstan and Uzbekistan. However, these even levels are owing to different population structures: the Central Asian countries have a higher proportion of children and lower proportion of persons at the age of 65 years and over, while in the Western European countries the proportion of children and persons at age 65+ are almost equal. 

**Figure 4:**
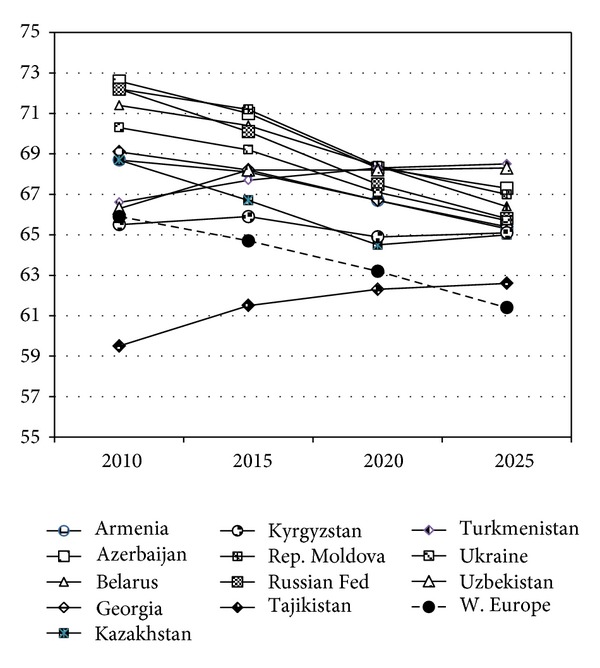
Changes in the size of the 15–64-years-old population, %, 2010–2025. Note: see Figure 3 (source: [16]).

Of special interest is the intertemporal dynamics of the relative size of this population group during the future 15 years (Figure 4). In the majority of the CIS countries, it is projected to steadily decline with the exception of Tajikistan, Turkmenistan and Uzbekistan, where it will be growing at least until 2025. Thus, by 2025, the levels of this indicator in the CIS countries will converge from the current range between 59.5% (Tajikistan) and 72.6% (Azerbaijan), to the more “homogeneous” range between 62.6% (Tajikistan) and 68.5% (Turkmenistan). In the Western European countries, the relative size of this population will undergo a rather steep decline from the current 65.9% to 61.4% in 2025. 

## 4. Challenges: Current Experiences of Active Ageing in the CIS Countries

This section reviews the situation in the CIS countries focusing on the three distinctive elements of the active ageing framework: employment, social participation, and independent living. 

### 4.1. Employment

**Figure 5:**
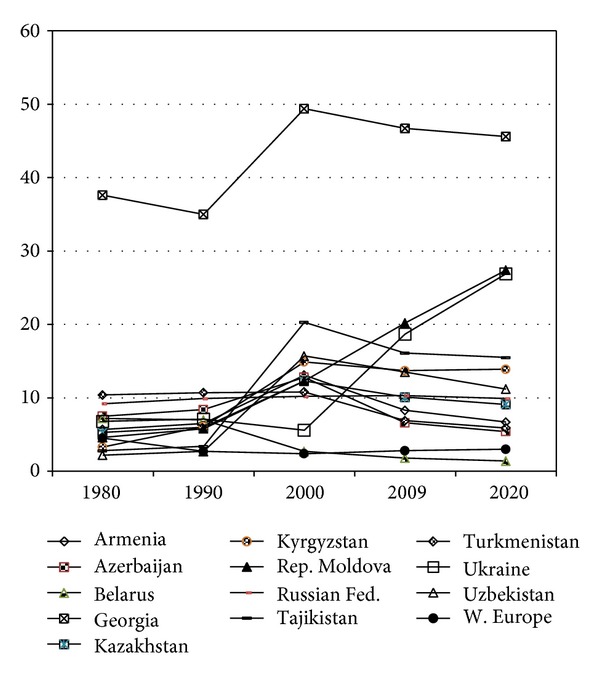
Labour force participation of persons at 65 years and over, %, 1980–2020 (source: [15]).

The labour force participation rates of persons of 65 years and over (LFP65+) is another good illustration of the diversity of the CIS countries. The differences between the countries are particularly apparent if this indicator is plotted against the time periods before and after the collapse of the USSR (see Figure 5). All CIS countries, except Belarus, had experienced a noticeable increase of LFP65+ during the decade 1990–2000. The highest increase was recorded in Tajikistan (16.9%), followed by Georgia (14.4%) and Uzbekistan (13%). In Ukraine, the comparable increase occurred in the following decade 2000–2009, when most of the CIS countries had undergone the reversal of the LFP65+ to the pretransitional levels. Moreover, the LFP65+ levels in Ukraine, as well as in the Republic of Moldova, are projected to grow during the decade leading to 2020. 

However, the situation concerning employment of persons of postretirement age in Ukraine, and most probably in many other CIS countries, can to a great extent be explained by a widespread employment of older age groups within the less prestigious jobs, and accordingly with minor competition from labour supply. Most of such jobs are available in the informal economy, but also at the state enterprises [18]. 

The distinctive patterns of the LFP65+ indicators are apparent in Belarus and Georgia. In Belarus, the levels of the LFP65+ have been the lowest among the CIS countries and very close to the corresponding figures in countries of the Western Europe. Note also that the LFP65+ in Belarus has been steadily declining since 1990. Meanwhile, the government of Belarus has recently reported the increase in the labour force participation of persons above the retirement age (55 years for women and 60 years for men) during the period of 2007–2010 [19]. In the Western European countries, where LFP65+ indicator has been much lower than in most CIS countries, it has also undergone an increase during the most recent decade 2000–2009. 

The Georgian case seems to be truly exceptional: not only this country had the highest level of LFP65+ before the transition, but it also recorded one of the highest degrees of increase in this indicator. Even in 2009, after some decline, LFP65+ in Georgia remained the highest among the CIS countries. The special case of Georgia can be explained by the possible influence of armed conflicts and border disputes that exacerbated the economic shock following the collapse of the USSR. As noted elsewhere, the effect of these factors could be seen most dramatically in the Caucasus region, where real GDP per capita virtually halved between 1991 and 1993 [20]. In contrast, Belarus before the transition (in 1989) had the highest level of GDP per capita among the Republics of the former Soviet Union, while between 1989 and 2002 the decline in its real GDP per capita was the smallest among the CIS countries. Thus, Belarus in 1999 had the lowest levels of poverty, including the level of absolute poverty, among the CIS countries [20]. Taken together, the above observations may indicate that in the CIS countries the high level of labour force participation of older persons, one of the experiences of active ageing, could reflect the individual adjustment to economic hardship rather than the informed response to policy incentives for continuing employment in older ages.

Any consideration of the employment situation of older persons in the CIS countries would be incomplete without recognizing that a significant, and in some countries even a predominant, portion of economy operates informally. Indeed, the size of the informal sector of economies in CIS countries in early 2000s might have varied from 30% to more than 60% of GDP [20]. In Kazakhstan, in 1999, the self-employed made up 45.1% of the total able-bodied population [21]. Many older workers who are employed work in the informal sector. This could be explained by the difficulties that older workers face in getting good jobs, and the possibilities of supplementing very low pension benefits with incomes from unregistered employment [22]. Again, the predominant role of economic necessity rather than incentives could also be seen behind this component of active ageing in the CIS countries. As noted in the 2012 national report of Ukraine on implementing the RIS/MIPAA, *“addressing the problem of the older people's efficient employment in Ukraine is more problematic compared to developed countries because of the archaic economic structure, prevailing traditional low-technology and labour intensive productions, widespread outdated technologies and equipment, harmful or arduous conditions of work, which by no means promotes long-term preservation of health and working capacity” *[18].

### 4.2. Social Participation

Data on the *social participation* of older persons in the CIS countries is scarce and unsystematic. The *content* and *forms* of *participation* of older persons as well as persons of other ages in the CIS countries have profoundly changed since the collapse of the Soviet Union. The essence of this change has been a shift from the collective to the individual forms of participation, predominantly in the family and also within the small networks of friends and acquaintances. As revealed in the 2008 wave of the European Values Study [23], the overwhelming majority (96%) of adults (18+ years old) in Russia put the family on the first place in the hierarchy of their values, followed by friends (85%) [23]. Participation in the family life and various contributions to its welfare, particularly through grandparenting, has substituted many other forms of participatory activities of older persons. While other forms of participation of older persons, as well as members of other age groups, have diminished after the collapse of the Soviet Union, this deeper attachment of older persons to the family life has been metaphorically referred to as “withdrawal to the family” [24].

Following the dismantling of the Soviet Union, the powerful top-down government-controlled organizations of older persons had been replaced by and in some cases transformed into the bottom-up initiatives and movements. During the Soviet period, there were long-established councils for war and labour veterans, operating at local municipal, regional, and national level, but these were essentially formal, politically managed agencies. During the years of transition, in some CIS countries, those councils have become more involved in the advocacy work on behalf of older persons, and in Ukraine some of them used to be represented in the parliament [25]. Moreover, in the Russian Federation and Ukraine, the parties of pensioners were established, and the Russian Party of Pensioners even entered the national parliament in 1999 [26]. As noted elsewhere, pensioner parties appeared to perform somewhat more successfully in the postcommunist Central and Eastern Europe than in the west European democracies [26]. The sporadic emergence of the new political and social organizations of older persons in the CIS countries during the post-communist transition can be driven by an abrupt collapse of welfare states and bottom-up attempts to replace them with self-help initiatives. Similar wave of new organizations of older persons was seen in Western Europe from 1970s in response to the “new politics of old age,” which was in turn created by the twin processes of population ageing and the contraction and reconfiguration of postwar welfare states [27]. 

Generally, in the CIS countries older persons are more politically active than the representatives of younger generations. In Kazakhstan, for instance, the share of the population that participates in the parliamentary elections increases with age, with 71.8% of those older than 65 voting, and only 52.4% in the age group 35–40 vote [21]. At the same time, older persons in Kazakhstan practically do not participate in the work of NGOs since these organizations are viewed negatively as supporting their respective organisers and not caring of their members [21]. Similar situation is noted in Tajikistan, where older persons after retirement are leaving the public life: only 5.7% took part in political organizations; 2.7% attended various clubs (e.g., sport clubs), 1.6% attended various exhibitions [28]. The situation is not much different in other CIS countries. In the Russian Federation, for instance, only 1% of persons at the age 55–69 years participated in the work of public organizations, and between 1% to 4% attended various cultural events [29].

People in the CIS countries also get involved very often in the volunteering work, including older persons. Interestingly, Turkmenistan was ranked number one among 153 world countries by the proportion of volunteers in a representative sample of individuals living across the country, who participated in the Gallup's World Poll survey in 2011 (see Table 2) [30]. Three other CIS countries from Central Asia (Kyrgyzstan, Tajikistan, and Uzbekistan), along with Belarus, were among the top twenty countries with the highest proportion of volunteers. Other seven CIS countries have been randomly distributed among various countries, including EU countries. There is a specific pattern in volunteering by the CIS persons in the upper age bracket of the Gallup's survey (50+ years old): the proportion of volunteers in this age group is lower than among the individuals in the total survey sample. No such uniform pattern is seen in the group of EU countries. 

### 4.3. Capacity for Independent Living: Health and Life Expectancy

In considering the component of independent living, we have focused on health as it captures best the independence aspect for older people and also this parameter has the most distinctive features and significance in the CIS countries. All CIS countries since 1991 have experienced the deterioration of health of their populations, albeit of different severity and during the different periods and length of time. The demographic and epidemiological patterns, and to some extent the medical, social, and economic mechanisms of this health decline, have been widely acknowledged and analysed in numerous publications [31–34]. The causes of the mortality crisis have been sorely investigated in the most affected Russian Federation, while the situation in other CIS countries has mostly escaped the attention of the international community. Below, we summarize the major demographic parameters of the health crisis in the CIS countries after the collapse of the Soviet Union, as it has implications for people's ability to live independently.

**Figure 6:**
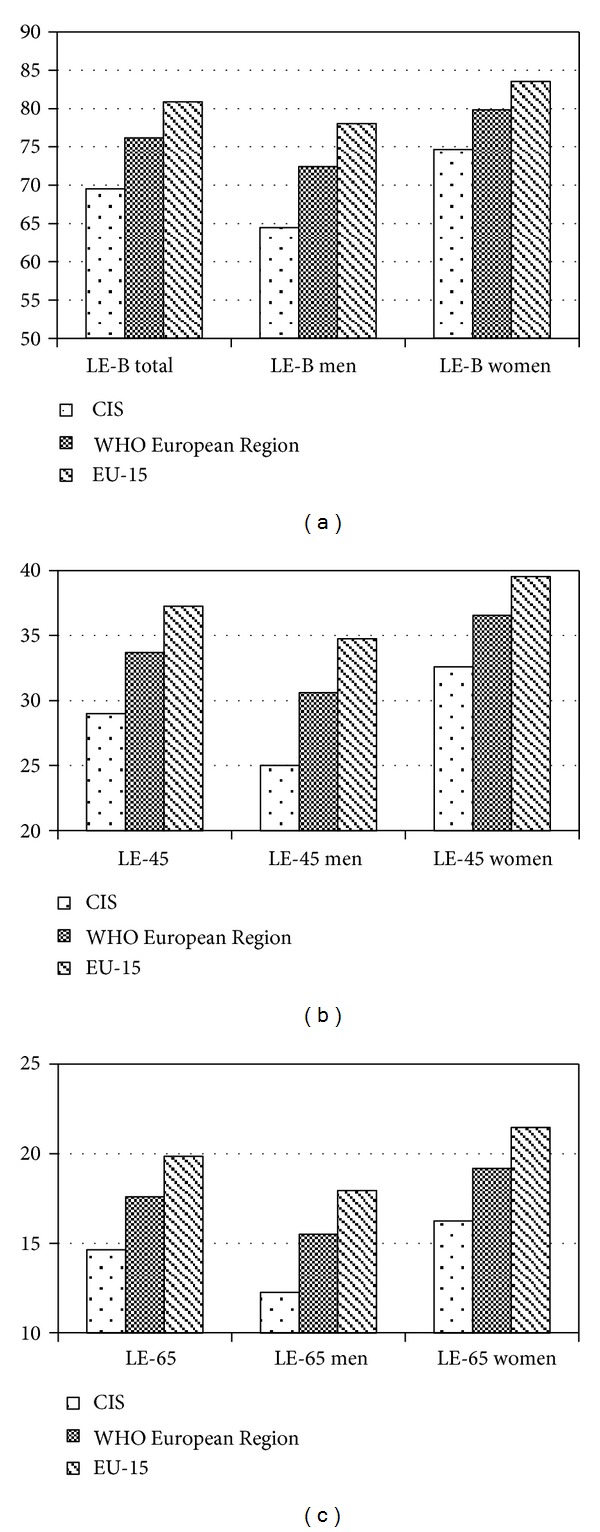
Life expectancy at birth (a) 45 years of age (b) and 65 years of age (c) 2008-2009 (Source: WHO Regional Office for Europe, European health for all database (HFA-DB) (http://data.euro.who.int/hfadb/shell_en.html). Note: the European region of WHO includes 53 countries: all European countries, all CIS countries, and also Israel and Turkey (http://www.who.int/about/regions/euro/en/index.html). EU-15 bloc of countries are: Austria, Belgium, Denmark, Finland, France, Germany, Great Britain, Greece, Ireland, Italy, Luxembourg, the Netherlands, Portugal, Spain, and Sweden.

Life expectancy at birth (LE-B) in CIS countries is significantly lower than in other countries of the European Region of WHO (see Figure 6(a)). The average total (men and women combined) LE-B in the CIS countries in 2008-2009 was 6.5 years (−9%) lower than the average LE-B in the European Region; moreover, for men this indicator was 8 years lower (−11%), and for women 5 years lower (−6%). The differences are even more apparent between the CIS countries and the 15 European countries that joined EU before May 2004 (EU-15): the total LE-B is 12 years (−15%) lower in CIS countries than in the EU-15 countries, men's LE-B is 14 years (−18%) lower, and LE-B for women is 9 years (−10%) lower. 

With age, the gap in absolute figures of life expectancy between the CIS and other European countries diminishes, while the differences in relative figures become even more apparent. The total life expectancy of persons of 45 years of age (LE-45) from the CIS countries (see Figure 6(b)) is 5 years (−15%) shorter than the average LE-45 in the WHO European Region; for 45 years old men this difference is 6 years shorter (−19%), and for 45 years old women 4 years shorter (−11%). Comparing to the citizens of EU-15, the citizens of the CIS countries of the same, 45 years of age, live 8 years less (−22%): men live 10 years less (−29%), and women 7 years less (−17%). 

The differences in life expectancy of 65 years old (LE-65) from the CIS countries and from the entire WHO European Region are the following: (−)3 years (−17%) in total LE-65; (−)4 years (−25%) in men's LE-65; (−)3 years (−16%) in women's LE-65 (Figure 6(c)). The differences with the EU-15 countries are again more pronounced: (−)5 years (−25%) in total LE-65; (−)6 years (−33%) in men's LE-65; (−)5 years (−24%) in women's LE-65. 

The decline in life expectancy in the CIS countries is one of the most obvious and tragic attributes of the transition years that followed the collapse of the USSR. Indeed, from 1990 to 1995, the five-year period during which the USSR ceased to exist, LE-B had dropped in almost all the CIS countries (see Figure 6). As was pointed elsewhere, mortality among Russian men in “productive age” is as high as it was 100 years ago [35]. The exceptions from the mortality crisis were Georgia and the Republic of Moldova, where it had not changed, and Kyrgyzstan, where LE-B had even increased slightly (by 0.2 years) comparing to the preceding five years (1985–1990). In absolute figures, the biggest decrease during that period was in the Russian Federation (−2.5 years) and Azerbaijan (−2.2 years). In Kazakhstan, the LE-B decline peaked during the next five year period (1995–2000) and was of the same level (−2.5 years) as in the Russian Federation during the preceding five years. 

**Figure 7:**
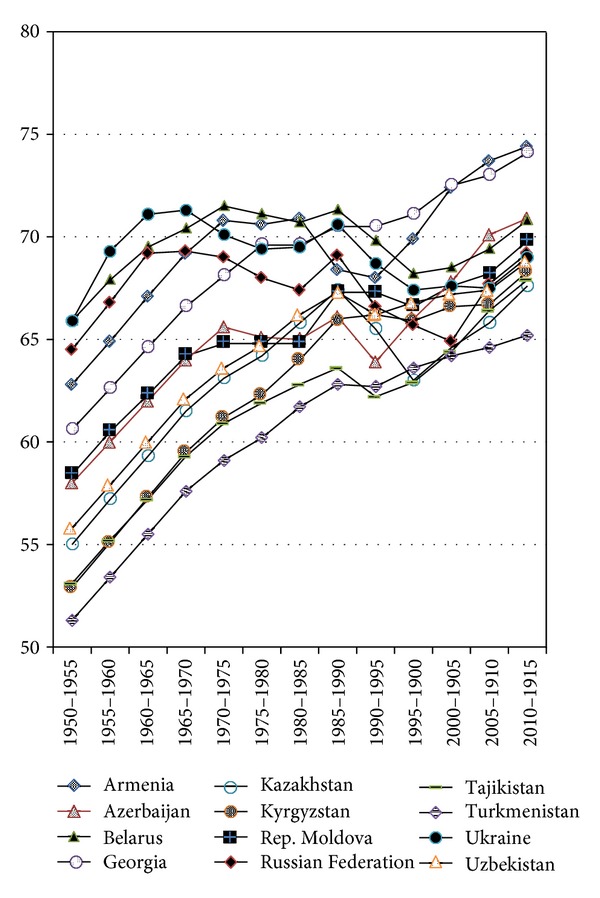
Life Expectancy at Birth in the CIS Countries, 1950–2015, medium variant (source: [16]).

The longest period of decline had been in the Russian Federation, where it had lasted for 15 years, until 2005; in Belarus, Kazakhstan, and Ukraine it had lasted for ten years, until 2000. During the periods of decline, LE-B dropped in Kazakhstan by 4.4 years; in the Russian Federation by 4.2 years; in Ukraine by 3.2 years; and in Belarus by 3.1 years. For men's LE-B the decline was even more manifest: (−)5.3 years in the Russian Federation; (−)4.9 years in Kazakhstan; (−)4.2 years in Ukraine; (−)4.1 years in Belarus. It should also be noted that in the recent years the life expectancy indicators in many CIS countries have shown some improvement (see Figure 7), while it will take longer time to see whether this trend is stable.

The investigators of the causes and mechanisms of the health crisis in the CIS countries, and particularly in the Russian Federation, have unanimously pointed to several factors, namely, heavy alcohol consumption, smoking, a high-fat diet, lack of leisure-time exercise, and hazardous working conditions [21, 34, 36]. Another important factor has been severe underfunding of public health care services that has put additional strain on the private out-of-pocket spending of the increasing impoverished segments of population in the CIS countries [22]. The total health expenditure in the Russian Federation in 2007-2008 was 5.3 percent of GDP, significantly below the levels observed in countries with similar per capita income [37]. In much less prosperous Tajikistan, this figure is even smaller: 3.5% of GDP [28]. The Russian Federation also spends less on health in per capita terms than other countries in the G-8 group and EU countries: 4.5 times less than Japan and 12 times less than USA. Not only health spending is low but also its outcome is low, too: health outcomes in Russia are similar to countries which spend 30–40% less on health. This is one of the evidences poor effectiveness and efficiency of the health care system organization and delivery in the Russian Federation [37].

The decline of the life expectancy and increase of the mortality in the CIS countries have been extraordinary following the collapse of the Soviet Union. It is worth pointing out that this decline was present, while not acknowledged, in the USSR long before the beginning of the transition (see Figure 7). This phenomenon was also pointed out by others [36]. Thus one can suppose that the shocks and pressures of the transition were the secondary triggering forces rather than the primary initiating mechanisms of the unprecedented peaceful time mortality crisis in the CIS countries. At this point, we can only speculate which of the great communist experiments in the USSR such as upturning the virgin soil and constructing the Baikal-Amur rail road might have disrupted the “normal” flow of the demographic process and brought the peoples of the soviet empire at the verge of the ethnosocial catastrophe. 

Another observation of a particular importance within the context of this paper is that during a few years following the antialcohol campaign of 1985, the mortality in many Republics of the USSR dropped and life expectancy noticeably increased (see Figure 7). The impact of this campaign points to the power of appropriate policy measures to overwhelm and prevent the negative social processes and thus to “normalize” the national demographic outlook. It was shown that behavioural factors are responsible for the 50% of difference in the mortality levels between the Russian Federation and the developed countries [38], and therefore combating those factors should be the focus of policy actions. Another promising example is the rapid decline of mortality in the countries of the Central and Eastern Europe after the collapse of the Berlin wall, which could be attributed to political, social, and economic changes [39]. A reasonable conclusion is therefore that policies for active ageing, and particularly those aimed at promoting healthy life styles, will be a good choice for the CIS countries.

## 5. Responses: Policies for Active Ageing in the CIS Countries

In spite of the demographic heterogeneity noted above, almost all governments of the CIS countries consider population ageing an important policy issue. That was the finding of the two surveys undertaken in 2007 and 2009 within the ongoing monitoring of national population policies by the United Nations Population Division [40, 41]. The results of the most recent 2009 survey revealed that all the CIS countries are concerned with the ageing of their populations (see Table 3). Out of the twelve CIS countries, ten consider population ageing to be a major concern and other two countries consider population ageing to be a minor concern. It is worth noting that in a short period of two years between the previous 2007 survey and the most recent 2009 survey, the governments of two countries, namely, Tajikistan and Turkmenistan, have raised the level of their concern of population ageing from “no concern” to “minor concern.” 

Ten out of twelve CIS countries consider the level of life expectancy at birth in their countries unsatisfactory, and only two, Armenia and Uzbekistan, are satisfied with their expected life expectancy. Armenia declared the “acceptable” level in 2009 survey as by that year it had achieved the highest level of life expectancy for men (70 years) and women (77 years) among the CIS countries. The second country whose government is satisfied with the life expectancy of its citizens is Uzbekistan, although the life expectancy in this country is relatively modest: 65 years for men and 71 years for women.

The CIS governments' views on the relative size of the working age population appear quite diverse: six out of the twelve countries expressed major concern about the size of their working age population; three countries expressed minor concern; the other three countries had no view on this issue. Interestingly, the countries that expressed the same level of concern belong to different stages of demographic transition: the major concern was expressed by Belarus, Kazakhstan, the Russian Federation, Tajikistan, Ukraine, and Uzbekistan; the minor concern was expressed by Armenia, Azerbaijan and Kyrgyzstan; Georgia, the Republic of Moldova, and Turkmenistan had “no view” on this population issue. 

The above evidence suggests that the views of the CIS governments on the ageing of their countries' population are somewhat disconnected from the demographic reality in their countries. It is difficult to detect apparent relations between the values of an indicator pertaining to a particular population issue (such as changes in life expectancy) and the government view of it. One possible explanation of this contradiction is that governments' views are more often influenced by the political considerations than anything else. Indeed, drawing a distinction between “policy” and “politics” in many ex-Soviet countries has been a daunting task as the old practice of the opinion-based policy formulations often prevails over the approach of the evidence-informed policy making. 

It is also necessary to mention that several CIS countries have been seriously concerned with the ongoing decline of their populations owing to the low fertility accompanied by the relatively high mortality. The largest absolute population declines among the CIS countries are expected in the Russian Federation, followed by Ukraine [22, 42]. Five CIS countries have registered annual decline of their populations in 2009: Belarus (−0.5%), Georgia (−1.1%), the Republic of Moldova (−1%), the Russian Federation (−0.4%), and Ukraine (−0.7%). Negative population growth has a particularly significant political connotation in these countries, fuelling the debates on the threat of depopulation and prompting policy measures aimed at raising the fertility rates. Practically all countries of the former Soviet Union address this challenge through pronatalistic policies and programmes. Population cohorts of higher ages, including older people and even people of working age, are traditionally overlooked from such policies. 

In this section we will also identify what policy responses to the challenges of population ageing are in place in the CIS countries. As the major source of information, we have used the national reports that the CIS countries submitted within the context of the review and appraisal of the Madrid International Plan of Action on Ageing (MIPAA). Since the adoption of MIPAA in 2002, the global review and appraisal exercise has been undertaken every five years: the first one was completed in 2007, and the second one is currently going on and will be completed in 2013. Within the first review and appraisal exercise, six CIS countries submitted their national reports: Armenia, Azerbaijan, Belarus, the Republic of Moldova, the Russian Federation, and Uzbekistan [43]. In the time of writing this paper, also six CIS countries submitted their reports for the second review and appraisal of MIPAA: Armenia, Belarus, the Republic of Moldova, the Russian Federation, Tajikistan, and Ukraine [44]. 

During the ten years after the Second World Assembly on Ageing in Madrid, all the reporting CIS countries had been elaborating and implementing various sectoral policies and programmes on ageing and older persons. Besides pursuing sectoral policies, several CIS countries, Armenia, Azerbaijan, Belarus, and the Republic of Moldova, have also undertaken measures aimed at *mainstreaming* the issues of ageing into their national development policies. 

Within the first implementation cycle (2002–2007), several policy priorities can be identified in CIS countries (see Table 4), with the three policy areas mentioned most often: *health and medical care* (6 countries) *social protection/income security*, and *integration and participation in societal life* (5 countries each).

During the second implementation cycle (2008–2012), the order of priorities had changed slightly. While *health and medical care* had remained among the top priorities (mentioned by five countries), two other policy areas, *social protection/income security*, and *social services,* had come at the top of the priority list as they had been mentioned by all of the six reporting countries. 

Even though not staying at the top of the list of priorities, another policy area, *social services*, has drawn much of the government attention. The essence of reforms of social services, including those targeting older persons, has been the replacement of categorized universal benefits by the targeted means-tested payments. In many cases, however, this proved to be a daunting task, including for administrative logistics, for the fact that numerous social benefits were administered by various government offices with vaguely defined entitlements, poorly monitored payments and weak, if any, interministerial coordination [45, 46].

Many CIS countries engaged in reforming the policies on ageing are challenged with the task of making them financially affordable. Hence, an increasing attention has been paid by some governments to the role of the family in care giving and reciprocal income security. The expectations are that this approach would save financial resources from being spent on much more expensive programmes of institutional care and formal income security. There is also a belief that the traditions of the extended family to care for its older members are still alive or could be revived in the Central Asian and Caucasian countries, and also in the Republic of Moldova [47]. At least three CIS countries, Armenia [48], Kazakhstan, and the Republic of Moldova [49], are implementing or considering policy measures focusing on the family as a major provider of services and resources for its members, including older persons [50]. For instance, in Armenia, as taking care for the older generation is among the most important values in the Armenian society, the social services support has been granted mainly to lonely old aged people (including those who have children but living alone and also those without children). 

The content of policy action varied within each policy area between different CIS countries. We have focused our instrumental analysis of those national policies which could be related to promoting the active ageing concept within the three dimensional framework of the EY2012 (as mentioned above): employment, social participation, and independent living. The findings are summarized in the Table 5.

### 5.1. Employment

Within the employment area, the EY2012 framework envisages measures aimed at *tackling early retirement*, *promoting flexible retirement*, and *providing incentives for extending working life*. Most of the CIS countries have undertaken various parametric reforms of their ex-Soviet defined-benefit pension schemes. Nine CIS countries, most recently (in 2011) Ukraine, have already increased the legal retirement age [46]. Similar measures have been under consideration in Belarus, the Russian Federation, and Uzbekistan. Notable exceptions from the prevailing parametric approach to reforming the pension systems has been Kazakhstan, where in 1998 a fully funded defined contribution scheme was introduced, as well as Kyrgyzstan and the Russian Federation, which have introduced some elements of the notional defined contribution scheme [17, 51]. Meanwhile, in the Russian Federation, the increase of retirement age and corresponding involvement of older workers in regular employment is not seen as a valuable option for improving the labour market situation [52]. 

Among the financial incentives for continuing employment introduced in the CIS countries are also various financial and judicial (antidiscrimination) measures. The pension legislation of the Russian Federation and Ukraine, as well as of some other CIS countries, provides for the right of working pensioners to receive their full pension along with the income from their work. In Russia, the size of pensions of the working pensioners is recalculated once every several years taking into account additional pension accruals.

In the recently (December 2011) drafted *Strategy on Ageing Issues and Social Protection of Older Persons*, the government of Armenia is planning to combat the age discrimination in employment and provide more favourable conditions for the older employees in the work place, for example, more flexible working schedule. 

In Belarus, the legislative measures to promote participation of older persons in the labour market have focused on preretirees and “young” (age 55–60) retirees. Such measures have aimed at adjusting labour market for making it more inclusive for various social groups, including people of older ages, by providing training programmes and psychological support to older job seekers, and coordinating the work of employment services and social services. 

In 2007, the Republic of Moldova adopted *The National Strategy on Employment Policies in the Republic of Moldova for the Period 2006–2020*. The medium term measures (2006–2010) aimed at reducing the early retirement; introducing incentives for flexible employment; promoting professional training programmes for preretirees; and organizing annual job market events for persons at pre-retirement age. The long-term measures (2011–2020) are focused on promoting policies for improving the work conditions, introducing incentives for a longer working career and preventing age discrimination in employment [49].

### 5.2. Social Participation

To facilitate social participation and promote active ageing in a wider perspective, measures are proposed in many CIS countries for the purpose of *encouraging voluntary activities*, *supporting informal carers*, and *recognizing contribution* of older persons to the various spheres of societal life.

Integration and participation in societal life was noted as a priority area in 2007 by five countries, and in 2012, by three countries (see Table 4). Again, as with other priority areas, the content and range of policy measures, either undertaken or planned, have varied between the countries. For instance, Azerbaijan, in its *State Programme to Increase Social Protection of Older Citizens* that was approved by the President in 2006, proclaimed that ensuring participation of older persons in socioeconomic and political life of society is among the major tasks of the *State Programme*. In the Republic of Moldova, the very limited public resources have been allocated for the cultural projects in various regions, and particularly in rural areas.

The Republic of Moldova has promoted intergenerational volunteering. As an example of bottom-up initiatives in the CIS countries, one worth-mentioning measure is the establishment in Belarus of self-help and mutual help groups for the reintegration of socially vulnerable older persons, particularly in small towns and villages. Similar initiatives exist in the Republic of Moldova and Ukraine and might be considered as an adjustment to the lack of access to or simply absence of appropriate governmental programmes.

Informal care givers are supported through the monthly allowances for family carers (in Belarus, the Russian Federation, and Ukraine) and also their inclusion for credits into the state pension schemes (Belarus, Ukraine). Training programmes for care volunteers and family carers are offered in Belarus, the Russian Federation, and Ukraine.

Governmental and nongovernmental programmes promoting positive images of older persons are reported by Armenia, Belarus, and the Russian Federation.

### 5.3. Policies Promoting Independent Living

Policies to promote independent living of older people include actions of promoting life-course preventive approach in health care; making the environment more age friendly; making transport more accessible for frail older persons.

As noted above, issues of health and medical care have been among the top priorities for policy actions on ageing in the CIS countries (Table 4). The policy measures in the area of health have ranged from improving the delivery of services to the measures of preventing old age associated diseases through promoting the healthy and active life styles. 

Many CIS countries see the task of increasing the life expectancy and lowering the mortality rates as a matter of their national security. In Belarus, for instance, the goals of decreasing the mortality and increasing the life expectancy at birth are included in the *National Programme on Demographic Security of the Republic of Belarus for 2011–2015*. The *Concept of the Long-Term (2008–2025) Socio-Economic Development of the Russian Federation* adopted in 2006, among its three goals, seeks to increase the life expectancy at birth to 70 years by 2015, and to 75 years by 2025. The latter figure, however, is considered unrealistic by some experts [52]. The *Programme of Economic Reforms in Ukraine for 2010–2014*, envisages measures, *inter alia*, for promoting the extension of life expectancy and, particularly, the period of active longevity, life duration, and improving the quality of life in the advanced ages.

It should be noted that practically all the CIS countries, as a legacy of the Soviet Union welfare state, possess the elements of a free health care system. Within such a system older persons in Belarus, for example, receive free annual medical examinations, and, in Armenia, free services in policlinics. Many CIS countries (Armenia, Azerbaijan, the Russian Federation, and Ukraine) have maintained or introduced new programmes for subsidizing the medicines for persons with disabilities and other categories of citizens. However, as mentioned above, severe under-funding of public health care systems forces private out-of-pocket spending, particularly in low income CIS countries [22].

Specialized geriatric services are offered only in a few CIS countries, namely, Belarus, the Republic of Moldova, the Russian Federation, and Ukraine, and geriatrics training is being provided in Belarus and Ukraine.

The *promotion of healthy life styles* has been undertaken through media campaigns (Belarus) and educational programmes (Ukraine). In Belarus, the *State Programme for Promoting Healthy Life Style* had been implemented during the periods 2002–2006 and 2007–2010, the *State Programme for Ensuring Sanitary and Epidemiological Well-Being*. In the Republic of Moldova, the availability of various programmes on raising awareness for a healthy life style has also been reported.

The issue of accessible and affordable transportation has been among the policy concerns and actions observed in Armenia, Azerbaijan, the Republic of Moldova, the Russian Federation, and Ukraine. In the Russian Federation and Ukraine, the corresponding measures have included financial subsidies towards providing free or discounted access to public transportation for certain categories of older persons, such as persons with disabilities and war veterans.

The barrier-free environment is an established policy area in Belarus, which has the *State Programme on Barrier-free Environment*. The safe living environment is also on the policy agenda in Armenia. The issue of affordable housing has been addressed through the state subsidies (Ukraine) and provision of social dwellings (Armenia, the Republic of Moldova). In Armenia, for instance, social dwellings for homeless lonely older persons have been developed since 2008.

## 6. Conclusions

CIS countries are undoubtedly a unique group of states, whose commonality and association is based primarily on their joint recent history. While they share the legacy of the Soviet past, they are different in many aspects: in their geographic, political, economic, social, and demographic spheres of life. In spite of all the differences, the legacy of the soviet past prevails in the structure and functioning of the CIS states, including the policy responses to various challenges. Population ageing challenges are among the most demanding, and by many countries in the region are considered a matter of social and economic stability. The linking issues of high mortality and migration are the top policy priorities, in practically all the CIS countries. 

Population ageing has been recognized as a matter of major concern by the majority of the CIS countries. In spite of this major-concern status assigned to population ageing by the governments of the respective countries, a comprehensive national strategy on ageing in these countries is a rare phenomenon. The existing sectoral policy and programmes on ageing are often a legacy of the pretransition era with modifications made on *ad hoc* basis. In some cases, more comprehensive and radical reforms were put aside owing to political uncertainties, lack of societal consensus and also financial instability. In some countries, the continuity of policy interventions has also been disrupted owing to the unfulfilled process of political transition and associated frequent government reshuffles.

Under such circumstances, policy measures of choice could be a consolidated approach aimed at addressing the major challenges and utilizing the major opportunities existing in the CIS countries. Among the major opportunities is the established yet slowly reformed and poorly financed government infrastructure on ageing and mainstreaming of ageing into national development strategies and other fundamental national policy action. Another resource is the growing potential of civil society organizations getting involved in various dimensions of policy action on ageing, not just in advocacy, but also in the service provision and in the policy advice. Equally important are the signs of improving capacity among researchers working on ageing who strive to collaborate at the international level, although additional support is required from the international community in building capacity for evidence-informed policymaking.

Once properly understood in the national terminology, the active ageing policy discourse can potentially become a promising approach in the CIS countries, as it allows them to address the challenges of population and individual ageing in a consolidated way by simultaneously tackling other demanding tasks of transitional societies: reforming the labour market, promoting social integration of older persons and intergenerational cohesion in a society, and addressing the health crisis. The guiding principles and actions identified under the European year for Active Ageing and Solidarity between Generations 2012 provide the right mix of inspiration and examples of best practices for the CIS countries.

## Figures and Tables

**Table 1:** Distribution (grouping) of CIS and EU countries by median age, 2009.

Median age (years)	CIS countries (rank)	EU countries (rank)
Above 35		Germany (2)
		Italy (3)
		Finland (4)
		Bulgaria (7)
		Austria (8)
		Slovenia (10)
		Greece (12)
		Belgium (13)
		Sweden (14)
		Portugal (15)
	Ukraine (27)	Denmark (16)
	Belarus (37)	Netherlands (17)
	Russian Federation (40)	Latvia (19)
	Georgia (47)	France (20)
		Spain (21)
		United Kingdom (22)
		Hungary (24)
		Lithuania (25)
		Estonia (26)
		Czech Republic (28) Luxembourg (29)
		Malta (32)
		Romania (36)
		Poland (41)
		Slovakia (48)
		Cyprus (52)

25–35	Republic of Moldova (56)	
	Armenia (65)	Ireland (58)
	Kazakhstan (77)	
	Azerbaijan (83)	

Below 25	Kyrgyzstan (109)	
	Turkmenistan (115)	
	Uzbekistan (117)	
	Tajikistan (146)	

Source: [15].

**Table 2:** CIS and EU countries ranked by the percentage of population volunteering time, 2011.

Volunteering time ranking	Country	Volunteering time (%)				
		Total population	Years old			
			15–24	25–34	35–49	50+
*1 *	*Turkmenistan *	*61 *	*64 *	*62 *	*60 *	*59 *
*4 *	*Tajikistan *	*44 *	*40 *	*49 *	*48 *	*38 *
*7 *	*Uzbekistan *	*41 *	*39 *	*40 *	*47 *	*37 *
13	Ireland	38	36	33	38	40
15	Netherlands	37	31	31	38	40
*16 *	*Kyrgyzstan *	*36 *	*37 *	*35 *	*43 *	*27 *
*18 *	*Belarus *	*35 *	*31 *	*36 *	*40 *	*34 *
21	Slovenia	34	45	32	30	34
28	Finland	30	21	33	40	29
*28 *	*Ukraine *	*30 *	*41 *	*34 *	*36 *	*19 *
31	Luxembourg	29	25	21	25	35
32	United Kingdom	28	33	21	28	29
37	Austria	27	24	31	27	26
37	France	27	22	18	27	31
42	Belgium	26	23	25	30	25
42	Cyprus	26	27	36	26	24
42	Germany	26	24	23	28	26
*47 *	*Kazakhstan *	*25 *	*31 *	*24 *	*26 *	*19 *
47	Malta	25	31	22	26	23
52	Denmark	24	17	18	24	28
*58 *	*Azerbaijan *	*23 *	*25 *	*26 *	*23 *	*19 *
*58 *	*Russian Fed. *	*23 *	*30 *	*23 *	*24 *	*18 *
*64 *	*Georgia *	*21 *	*19 *	*19 *	*29 *	*18 *
*64 *	*Rep. Moldova *	*21 *	*20 *	*33 *	*24 *	*12 *
78	Czech Rep.	18	18	22	21	14
78	Latvia	18	13	19	23	15
78	Spain	18	18	18	20	16
92	Slovakia	16	14	19	15	16
99	Estonia	15	13	20	16	13
101	Italy	14	16	12	18	11
110	Poland	13	17	17	10	11
*119 *	*Armenia *	*11 *	*12 *	*14 *	*12 *	*8 *
119	Sweden	11	10	15	12	11
127	Portugal	10	9	17	9	9
133	Lithuania	9	11	3	14	7
135	Hungary	8	5	12	8	9
146	Bulgaria	5	6	3	10	4
146	Romania	5	10	4	3	4
153	Greece	3	2	2	6	2

“Volunteering time in %” is a proportion of respondents that have positively responded to the question: “Have you volunteered your time to an organization (in the past month)?”

Source: [30].

**Table 3:** Views of the CIS governments on selected population issues, 2009.

Country	Population Issue		
	Ageing	Life expectancy at birth	Size of working age population
Armenia	Major concern	*Acceptable *	*Minor concern *
Azerbaijan	Major concern	Unacceptable	*Minor concern *
Belarus	Major concern	Unacceptable	Major concern
Georgia	Major concern	Unacceptable	—
Kazakhstan	Major concern	Unacceptable	Major concern
Kyrgyzstan	*Minor concern *	Unacceptable	*Minor concern *
Republic of Moldova	*Minor concern *	Unacceptable	—
Russian Federation	Major concern	Unacceptable	Major concern
Tajikistan	*Minor concern *	Unacceptable	Major concern
Turkmenistan	*Minor concern *	Unacceptable	—
Ukraine	Major concern	Unacceptable	Major concern
Uzbekistan	Major concern	*Acceptable *	Major concern

(—): no view expressed.

Source: [41].

**Table 4:** Areas of policy priorities on ageing in CIS countries, 2007, 2012.

Priority area	2007		2012	
	Number of countries quoted the priority area	Quoting countries	Number of countries quoted the priority area	Quoting countries
Health and medical care	6	AM, BY, AZ, BY, MD, RU	5	AM, BY, MD, RU, UA
Social protection/income security	5	AM, AZ, BY, MD, RU	6	AM, BY, MD, RU, TJ, UA
Integration and participation in societal life	5	AM, AZ, BY, MD, RU	3	AM, BY, RU
Rights of older persons/antiage discrimination	4	AZ, MD, RU, UZ	3	AM, MD, UA
Social services	4	AZ, BY, MD, RU	6	AM, BY, MD, RU, TJ, UA
Developing (strengthening) institutional infrastructure	3	AM, AZ, RU	1	MD
Labour market measures	3	MD; BY; RU	2	AM, BY, MD
Social care, including long-term care	2	AM, RU	4	AM, MD, TJ, UA
Intergenerational cohesion (solidarity)	2	MD, UZ	3	BY, MD, RU
Promoting positive image of ageing and older persons in society	2	AM, RU	2	BY, RU
Sociocultural needs	2	AZ, AM	4	AM, MD, RU, UA
Research on ageing	2	AZ, RU	3	BY, RU, UA
Providing secure and affordable living environment	2	AM, UZ	4	AM, BY, MD, UA
Affordable and accessible transportation	2	AM, MD	2	RU, UA
Promoting life-long learning	1	MD	4	AM, BY, RU, UA
Adjusting public finance policy to demographic ageing	—	—	2	RU, UA

Abbreviations used: Armenia (AM); Azerbaijan (AZ); Belarus (BY); Republic of Moldova (MD); Russian Federation (RU); Tajikistan (TJ); Ukraine (UA); Uzbekistan (UZ). Source: [43, 44].

**Table 5:** Policy measures promoting active ageing in the CIS countries.

EU Framework for the EY2012	Approaches to promoting active ageing	Policy measures by CIS countries 2002–2007	Policy measures by CIS countries 2008–2012
Employment	Tackling early retirement	(i) Increasing age of mandatory retirement (AM 1996, AZ, TJ 2005)	(i) No compulsory retirement (BY) (ii) Increasing age of mandatory retirement (UA 2011)
	Promoting flexible retirement	(i) Employment assistance and retraining (BY, MD, RU) (ii) Coordination of employment services and social services (BY) (iii) Psychological support to older job seekers (RU)	(i) Favourable and flexible conditions (AM, UA) (ii) Employment assistance (RU)
	Providing incentives for extending working life	(i) Support to preretirees and “young” retirees (BY)	(i) Combating discrimination (AM; RU) (ii) Competitiveness in the labour market (BY) (iii) Incentives for extending working life (RU, UA)

Participation	Encouraging voluntary activities	—	(i) Help and self-help groups (BY, MD, UA) (ii) Intergenerational volunteering (MD)
	Supporting informal carers	(i) Monthly allowances for family carers (BY, RU) (ii) Training care volunteers and family members (RU, UA) (iii) Pension coverage (BY)	(i) Training of care volunteers and family members (BY, UA) (ii) Monthly allowances for family carers (UA) (iii) Pension coverage (UA)
	Recognizing contribution	(i) Promoting positive image (AM, RU)	(i) Promoting positive image (BY) (ii) Recognizing contribution (RU)

Independent living	Promoting life-course preventive approach in health care	(i) Free medical care (AZ, BY, RU) (ii) Free service in policlinics (AM) (iii) Discounts for medicines (AM, AZ, RU) (iv) State programmes for promoting healthy life style (BY) (v) Geriatrics services (RU) (vi) Geriatrics training (BY)	(i) Decreasing mortality, increasing LEB (BY, RU) (ii) Free medical care (AM, BY, RU, UA) (iii) Annual medical examinations (BY) (iv) Geriatrics training (BY, UA) (v) Healthy life styles in media (BY) and education (UA) (vi) The National Program for Healthy Lifestyle Promotion 2007–2015 (MD) (vii) Preventing disability (MD)
	Making transport more accessible	(i) Accessible and affordable transportation (AM, AZ, MD, RU)	(i) Accessible and affordable transportation (RU, UA)
	Making the environment more age friendly	(i) Safe living environment (AM; BY) (ii) Barrier free environment (BY)	(i) Social dwellings/housing (AM, MD) (ii) Barrier free environment (BY) (iii) Housing subsidies (UA)

Source: [43, 44]. For country abbreviations, see the footnote of Table 4.
